# Smoking Behaviors and Prognosis in Patients With Non–Muscle-Invasive Bladder Cancer in the Be-Well Study

**DOI:** 10.1001/jamanetworkopen.2022.44430

**Published:** 2022-11-30

**Authors:** Marilyn L. Kwan, Reina Haque, Kelly C. Young-Wolff, Valerie S. Lee, Janise M. Roh, Isaac J. Ergas, Zinian Wang, Kimberly L. Cannavale, Christine B. Ambrosone, Ronald K. Loo, David S. Aaronson, Charles P. Quesenberry, Lawrence H. Kushi, Li Tang

**Affiliations:** 1 Division of Research, Kaiser Permanente Northern California, Oakland; 2 Department of Research & Evaluation, Kaiser Permanente Southern California, Pasadena; 3 Department of Health Systems Science, Kaiser Permanente Bernard J. Tyson School of Medicine, Pasadena, California; 4 Department of Cancer Prevention and Control, Roswell Park Comprehensive Cancer Center, Buffalo, New York; 5 Department of Urology, Kaiser Permanente Downey Medical Center, Downey, California; 6 Department of Urology, Kaiser Permanente Oakland Medical Center, Oakland, California

## Abstract

**Importance:**

Tobacco smoking is an established risk factor associated with bladder cancer, yet its impact on bladder cancer prognosis is unclear.

**Objective:**

To examine associations of use of tobacco (cigarettes, pipes, and cigars), e-cigarettes, and marijuana with risk of recurrence and progression of non–muscle-invasive bladder cancer (NMIBC) and to explore use of smoking cessation interventions.

**Design, Setting, and Participants:**

The Be-Well Study is a prospective cohort study of patients with NMIBC diagnosed from 2015 to 2019 and followed-up for 26.4 months in the Kaiser Permanente Northern and Southern California integrated health care system. Eligibility criteria were age at least 21 years, first NMIBC diagnosis (stages Ta, Tis, or T1), alive, and not in hospice care. Exclusion criteria were previous diagnosis of bladder cancer or other cancer diagnoses within 1 year prior to or concurrent with NMIBC diagnosis. Data were analyzed from April 1 to October 4, 2022.

**Exposures:**

Use of cigarettes, pipes, cigars, e-cigarettes, and marijuana was reported in the baseline interview. Use of smoking cessation interventions (counseling and medications) was derived from electronic health records.

**Main Outcomes and Measures:**

Hazard ratios (HRs) and 95% CIs of recurrence and progression of bladder cancer were estimated by multivariable Cox proportional hazards regression.

**Results:**

A total of 1472 patients (mean [SD] age at diagnosis, 70.2 [10.8%] years; 1129 [76.7%] male patients) with NMIBC were enrolled at a mean (SD) of 2.3 (1.3) months after diagnosis, including 874 patients (59.4%) who were former smokers and 111 patients (7.5%) who were current cigarette smokers; 67 patients (13.7%) smoked pipes and/or cigars only, 65 patients (4.4%) used e-cigarettes, 363 patients (24.7%) used marijuana. Longer cigarette smoking duration and more pack-years were associated with higher risk of recurrence in a dose-dependent manner, with the highest risks for patients who had smoked for 40 or more years (HR, 2.36; 95% CI, 1.43-3.91) or 40 or more pack-years (HR, 1.97; 95% CI, 1.32-2.95). There was no association of having ever smoked, being a former or current cigarette smoker, and years since quit smoking with recurrence risk. No associations with pipes, cigars, e-cigarettes, or marijuana were found. Of 102 patients offered a smoking cessation intervention, 57 (53.8%) received an interventions after diagnosis, with female patients more likely than male patients to engage in such interventions (23 of 30 female patients [76.7%] vs 34 of 76 male patients [44.7%]; *P* = .003).

**Conclusions and Relevance:**

These findings suggest that longer duration and more pack-years of cigarette smoking were associated with higher risk of NMIBC recurrence. Cigarette smoking remains a critical exposure before and after diagnosis in survivors of NMIBC.

## Key Points

**Question:**

Are tobacco smoking and marijuana use associated with recurrence risk in patients with non–muscle-invasive bladder cancer (NMIBC)?

**Findings:**

In this cohort study of 1472 adults with NMIBC, two-thirds of whom were former or current cigarette smokers at diagnosis, longer duration and increasing pack-years of cigarette smoking were associated with higher recurrence risk in an exposure-response manner, with up to twice the risk for 40 or more years of smoking and 40 or more pack-years. Pipe, cigar, marijuana, and e-cigarette use were not associated with recurrence risk.

**Meaning:**

These findings suggest that duration and lifetime pack-years of cigarette smoking were associated with NMIBC recurrence risk in a dose-dependent fashion.

## Teaser

This cohort study assesses associations of tobacco, e-cigarette, and marijuana use with recurrence risk in patients with non–muscular invasive bladder cancer.

## Introduction

In 2022, approximately 81 000 people in the US will be diagnosed with bladder cancer, making it the 4th most common cancer in men and 11th most common in women.^1^ Tobacco smoking is the most important modifiable factor associated with bladder cancer risk,^2,3^ accounting for nearly half (47%) of all cases in the US.^1^

Previous studies have shown more than 3-fold increased risk of bladder cancer in current tobacco smokers and 2-fold increased risk in former smokers compared with nonsmokers.^4^ Furthermore, a 2016 meta-analysis^5^ of 89 observational studies from the last 50 years reported that smoking is associated with the highest risk of bladder cancer compared with any other environmental or occupational risk factor. Increasing risk of bladder cancer was also observed with increasing number of cigarettes smoked, with peak risk noted at approximately 15 cigarettes smoked per day.

However, the impact of tobacco smoking on bladder cancer prognosis is unclear. Studies of patients with non–muscle-invasive bladder cancer (NMIBC), which is the most common type of bladder cancer and represents approximately 75% of cases,^6^ have reported mixed results on the impact of lifetime smoking behavior on cancer outcomes. Some studies have found that current, heavy, and long-term prediagnosis smoking is associated with worse recurrence-free survival,^7,8,9,10,11^ while other studies have found no association.^12,13,14^ All analyses were limited by relatively small sample sizes, inconsistent exposure assessment, and retrospective study design.

Public awareness of bladder cancer as a tobacco-related disease is poor compared with other tobacco-related conditions.^15,16^ In addition, urologists do not necessarily believe that smoking cessation plays any role in the clinical management of bladder cancer.^17^ Patient education and improved clinician recognition of the importance of smoking cessation in disease management may help improve outcomes.^18^ However, to our knowledge, no prior studies have evaluated smoking cessation interventions in patients with bladder cancer in an integrated health care setting.

Therefore, we conducted a comprehensive analysis of smoking behavior at cancer diagnosis and its association with prognosis in one of the largest prospective cohorts of newly diagnosed patients with NMIBC. Our primary goal was to quantify risk associations of tobacco smoking (cigarette, pipe, and cigar), e-cigarette, and marijuana use with NMIBC recurrence and progression. Our secondary, exploratory goal was to describe smoking cessation interventions in patients with NMIBC diagnosed and treated in an integrated health system.

## Methods

This cohort study was approved by ethics boards at all study sites. All participants provided informed consent by agreeing to participate in the survey. This report follows the Strengthening the Reporting of Observational Studies in Epidemiology (STROBE) reporting guideline for observational studies.

### Study Design

The Be-Well Study is a prospective cohort study of patients with NMIBC, examining the associations of nutritional, lifestyle, and genetic factors in bladder cancer treatment and outcomes.^19^ Patients were recruited from Kaiser Permanente Northern California (KPNC) and Southern California (KPSC) from 2015 to 2019. Eligibility criteria included age 21 years or older, first diagnosis of NMIBC, alive, and not in hospice care. Exclusion criteria included previous diagnosis of bladder cancer or other cancer diagnoses within 1 year prior to or concurrent with NMIBC diagnosis.

Patients with newly diagnosed NMIBC were identified from automated daily scans of electronic pathology records using preselected Systematized Nomenclature of Medicine-Clinical Terms indicative of malignant bladder cancer at KPNC and from electronic extraction of pathology reports using text string searches for *bladder* or *urothelial* on a semimonthly schedule at KPSC. Study eligibility and patient notification of their diagnosis were confirmed from review of electronic health records (EHR). Study enrollment was defined by completion of the baseline interview.

### Baseline Data Collection

The baseline interview included the main questionnaire and a food frequency questionnaire^20,21^ administered to the participant via computer-assisted telephone interview. The main questionnaire covered bladder cancer diagnosis and medical history, bladder function and quality of life, lifestyle factors, environmental and occupational exposures, and sociodemographic characteristics. Race and ethnicity categories were defined by the investigator and included the analysis as an important potential confounder. Race and ethnicity categories were Asian, Black, Hispanic, White, and other (including American Indian, Alaska Native, and Pacific Islander).

### Tobacco and Marijuana Exposure

Participants reported their history of smoking cigarettes and using pipes, cigars, e-cigarettes, and marijuana at the baseline interview. Questions included ever use, current use, age started and stopped, duration excluding quit periods, and mean among smoked or vaped per day. Mode of marijuana exposure (ie, smoking, edible, other) was not queried. Questions are available in eTable 1 in Supplement 1.

For analyses, cigarette smoking was categorized as never, former, and current, and by duration in years (<10, 10-19, 20-29, 30-39, and ≥40 years), intensity in cigarettes per day (1-9, 10-19, 20-29, 30-39, and ≥40 cigarettes per day), pack-years (<10, 10-19, 20-29, 30-39, and ≥40 pack-years), and years since quit smoking based on age at NMIBC diagnosis (stopped ≥20 years ago, 10-19 years ago, 1-9 years ago, and <1 year ago). For analyses of progression risk only, categories for duration in years (<20, 20-39, ≥40 years), intensity (1-19, 20-29, and ≥30 cigarettes per day), and pack-years (<20, 20-39, and ≥40 pack-years) were modified to accommodate small cell sizes. For pipes, cigars, and e-cigarettes, use was categorized as never or ever, while marijuana use was categorized as never, former, or current. A combined marijuana and any tobacco use variable, including cigarette smoking, was also created (no tobacco or marijuana use, tobacco use only, marijuana use only, tobacco and marijuana use).

### Smoking Cessation Interventions

At KPNC, smoking cessation interventions are offered via in-person classes, counseling, and individual telephone coaching at no cost to the patient. Health educators conduct classes and in-person counseling and focus on providing education about nicotine addiction, discussing tobacco cessation medications, and developing a quit plan. Individual telephone coaching sessions are also available with wellness coaches trained in motivational interviewing.^22^

Data on smoking cessation interventions at KPNC were obtained from the EHR beginning 6 months before until 12 months after NMIBC diagnosis for a sample of 68 current cigarette smokers and 38 recent quitters (within ≤1 year before their diagnosis) as determined from the baseline interview. Smoking cessation counseling (health education and wellness coaching) was found in encounter, referral, and physician notes. Tobacco cessation medication use (bupropion, varenicline, and nicotine replacement therapy, including inhalers, sprays, patch, gum, and lozenges) was extracted from outpatient pharmacy tables.^23^

### Covariates

Data on sociodemographic characteristics, body mass index (BMI; calculated as weight in kilograms divided by height in meters squared), alcohol consumption, dietary folate, environmental and chemical exposures, and occupation involving environmental and chemical exposures came from the baseline interview. Pathologic and clinical characteristics, including cancer stage (Ta, T1, or Tis) and grade (low or high grade), were obtained from the KPNC and KPSC cancer registries,^24^ and information on use of intravesical chemotherapy (mitomycin) and immunotherapy (Bacillus Calmette-Guerin) were extracted from the 24-month EHR review. The American Urological Association risk stratification category was calculated for each patient.^25^

### Outcomes Ascertainment

A comprehensive EHR review was completed by trained medical records abstractors for recurrence and progression events at the 24-month follow-up, given most NMIBC recurrences occur within 24 months of initial diagnosis.^26,27^ Events were ascertained primarily from pathology and cytology reports. Recurrence was defined as a newly detected bladder tumor at least 3 months after previous negative or inconclusive cystoscopy findings. Progression, a subset of recurrence, was defined as tumor progression by stage or grade within non–muscle-invasive categories (Ta to T1, Ta or T1 to Tis, or low to high grade) or into muscle-invasive disease (stage ≥T2).

### Statistical Analysis

Distributions of smoking behaviors were summarized through means, SDs, and ranges for continuous variables, and frequencies and percentages for categorical variables. *P* values to detect differences between groups were 2-sided and were calculated by Pearson χ^2^ test or Fisher exact test.

Hazard ratios (HRs) and 95% CIs estimating the association of smoking behaviors with developing recurrence and progression combined and progression only were calculated using multivariable Cox proportional hazards regression.^28^ Follow-up was calculated as time since date of NMIBC diagnosis to first date of recurrence or progression, loss to follow-up, death, or 24-month EHR review. Two models were created for each of the study outcomes. Model 1 was adjusted for preselected covariates, including age at diagnosis, sex, race and ethnicity, educational attainment, household income, BMI at diagnosis, cancer stage and grade, chemotherapy, immunotherapy, and American Urological Association risk category. Model 2 included the same covariates in model 1, plus alcohol intake, dietary folate, overall chemical or environmental exposures, and occupation involving chemical or environmental exposures. In addition, the cigarette smoking models were adjusted for other tobacco, e-cigarette, and marijuana use. The pipe, cigar, e-cigarette, and marijuana use models were adjusted for tobacco and e-cigarette use as applicable. *P* values for trends for cigarette smoking duration, intensity, and pack-years were calculated by treating the variables as continuous variables. The proportional hazards assumption was tested using Schoenfeld residuals,^29^ and no evidence of violations across the covariates were found. Statistical significance was considered as *P* < .05. Analyses were conducted in SAS statistical software version 9.4 (SAS Institute). Data were analyzed from April 1 to October 4, 2022.

## Results

The cohort included 1472 patients (mean [SD] age at diagnosis, 70.2 [10.8%] years; 1129 [76.7%] male patients) with NMIBC, including 72 Asian patients (4.9%), 81 Black patients (5.5%), 128 Hispanic patients (8.7%), and 1165 White patients (79.1%) (Table 1). Approximately half of all patients were diagnosed with low-grade Ta (632 patients [42.9%]) and T1 (43 patients 2.9%) disease. The remainder were diagnosed with high-grade Ta (352 patients [23.9%]) or T1 (370 patients [25.1%]) disease or Tis (63 patients [4.3%]).

**Table 1.** Characteristics of Be-Well Study Participants by Cigarette Smoking Behavior

Characteristic	Participants, No. (%)				*P* value^a^
	Total (N = 1472)	Cigarette smoking status			
		Never (n = 487)	Former (n = 874)	Current (n = 111)	
**Sociodemographic and clinical characteristics**					
Cancer stage and grade					
LG Ta	632 (42.9)	235 (48.3)	350 (40.1)	47 (42.3)	.21
LG T1	43 (2.9)	10 (2.1)	28 (3.2)	5 (4.5)	
HG Ta	352 (23.9)	100 (20.5)	225 (25.7)	27 (24.3)	
HG T1	370 (25.1)	117 (24.0)	226 (25.9)	27 (24.3)	
Tis	63 (4.3)	23 (4.7)	36 (4.1)	4 (3.6)	
Other	12 (0.8)	2 (0.4)	9 (1)	1 (0.9)	
Concomitant CIS					
No	1331 (90.4)	442 (90.8)	783 (89.6)	106 (95.5)	.58
Yes with Ta disease	46 (3.1)	14 (2.9)	30 (3.4)	2 (1.8)	
Yes with T1 disease	68 (4.6)	22 (4.5)	43 (4.9)	3 (2.7)	
CIS only	27 (1.8)	9 (1.9)	18 (2.1)	0	
Chemotherapy					
No	896 (60.1)	304 (62.4)	530 (60.6)	62 (55.9)	.43
Yes	576 (39.1)	183 (37.6)	344 (39.4)	49 (44.1)	
Immunotherapy					
No	704 (47.8)	251 (51.5)	404 (46.2)	49 (44.1)	.12
Yes	768 (52.2)	236 (48.5)	470 (53.8)	62 (55.9)	
Sex					
Male	1129 (76.7)	359 (73.7)	691 (79.1)	79 (71.2)	.03
Female	343 (23.3)	128 (26.3)	183 (20.9)	32 (28.8)	
Race and ethnicity					
Asian	72 (4.9)	42 (8.6)	29 (3.3)	1 (0.9)	<.001
Black	81 (5.5)	20 (4.1)	52 (6.0)	9 (8.1)	
Hispanic	128 (8.7)	42 (8.6)	75 (8.6)	11 (9.9)	
White	1165 (79.1)	379 (77.8)	699 (80.0)	87 (78.4)	
Other^b^	26 (1.8)	4 (0.8)	19 (2.2)	3 (2.7)	
Age, y					
<50	64 (4.3)	33 (6.8)	25 (2.9)	6 (5.4)	<.001
50-64	356 (24.2)	141 (29.0)	173 (19.8)	42 (37.8)	
65-79	773 (52.5)	225 (46.2)	491 (56.2)	57 (51.4)	
≥80	279 (19.0)	88 (18.1)	185 (21.2)	6 (5.4)	
Mean (SD)	70.2 (10.8)	68.4 (11.8)	71.6 (10.1)	66.9 (9.4)	<.001
BMI at diagnosis					
≤24.9	388 (26.4)	152 (31.2)	198 (22.7)	38 (34.2)	<.001
25.0-29.9	598 (40.6)	201 (41.3)	357 (40.9)	40 (36.0)	
≥30.0	486 (33.0)	134 (27.5)	319 (36.5)	33 (29.7)	
Mean (SD)	28.6 (5.7)	28.1 (5.7)	29.0 (5.6)	27.5 (5.6)	.001
Marital status					
Never married	73 (5.0)	26 (5.3)	40 (4.6)	7 (6.3)	<.001
Married or living as married	1026 (69.7)	372 (76.4)	595 (68.1)	59 (53.2)	
Separated or divorced	201 (13.6)	41 (8.4)	128 (14.7)	32 (28.8)	
Widowed	162 (11.0)	43 (8.8)	106 (12.1)	13 (11.7)	
Unknown	10 (0.7)	5 (1.0)	5 (0.6)	0	
Educational attainment					
≤High school	251 (17.1)	49 (10.1)	165 (18.9)	37 (33.3)	<.001
Some college	504 (34.2)	134 (27.5)	326 (37.3)	44 (39.6)	
College graduate	393 (26.7)	160 (32.9)	214 (24.5)	19 (17.1)	
Postgraduate	305 (20.7)	134 (27.5)	160 (18.3)	11 (9.9)	
Unknown	19 (1.3)	10 (2.1)	9 (1.0)	0	
Household income					
<$60 000	458 (31.1)	106 (21.8)	299 (34.2)	53 (47.8)	<.001
$60 000-$99 999	346 (23.5)	117 (24.0)	206 (23.6)	23 (20.7)	
≥$100 000	494 (33.6)	216 (44.4)	261 (29.9)	17 (15.3)	
Unknown	174 (11.8)	48 (9.9)	108 (12.4)	18 (16.2)	
Alcohol consumption^c^					
None	335 (22.8)	108 (22.2)	203 (23.2)	24 (21.6)	.31
Rare	237 (16.1)	90 (18.5)	135 (15.5)	12 (10.8)	
Occasional	236 (16.0)	88 (18.1)	131 (15.0)	17 (15.3)	
Regular	373 (25.3)	111 (22.8)	235 (26.9)	27 (24.3)	
Unknown	291 (19.8)	90 (18.5)	170 (19.5)	31 (27.9)	
Dietary folate, mean (SD), µg	301.64 (148.59)	311.22 (153.70)	294.21 (142.59)	319.50 (170.80)	.10
Overall environmental and chemical exposures^d^					
Never	472 (32.1)	173 (35.5)	276 (31.6)	23 (20.7)	.009
Ever	1000 (67.9)	314 (64.5)	598 (68.4)	88 (79.3)	
Occupation involving environmental and chemical exposures^e^					
Never	958 (65.1)	355 (72.9)	539 (61.7)	64 (57.7)	<.001
Ever	514 (34.9)	132 (27.1)	335 (38.3)	47 (42.3)	
**Cigarette smoking characteristics**					
Duration (in smokers), y					
<10	NA	NA	162 (18.5)	0	NA
10-19	NA	NA	191 (21.9)	0	
20-29	NA	NA	202 (23.1)	5 (4.5)	
30-39	NA	NA	177 (20.3)	12 (10.8)	
≥40	NA	NA	141 (16.1)	94 (84.7)	
Mean (SD) [range]	NA	NA	23.8 (14.5) [0-76.0]	50.0 (10.6) [22.1-76.9]	
Median (IQR)	NA	NA	23.0 (12.0-34.0)	49.7 (43.7-58.2)	
Intensity (in smokers), cigarettes/d					
1-9	NA	NA	151 (17.3)	48 (43.2)	NA
10-19	NA	NA	198 (22.7)	38 (34.2)	
20-29	NA	NA	325 (37.2)	19 (17.1)	
30-39	NA	NA	88 (10.1)	3 (2.7)	
≥40	NA	NA	99 (11.3)	3 (2.7)	
Mean (SD) [range]	NA	NA	19.4 (12.9) [0-80.0]	11.6 (8.4) [1.0-40.0]	
Median (IQR)	NA	NA	20.0 (10.0-20.0)	10.0 (5.0-16.0)	
Pack-years (in smokers)					
<10	NA	NA	251 (28.7)	22 (19.8)	NA
10-19	NA	NA	164 (18.8)	25 (22.5)	
20-29	NA	NA	134 (15.3)	21 (18.9)	
30-39	NA	NA	120 (13.7)	11 (9.9)	
≥40	NA	NA	197 (22.5)	32 (28.8)	
Mean (SD) [range]	NA	NA	25.6 (23.3) [0-156.0]	29.5 (23.6) [1.4-132.6]	
Median (IQR)	NA	NA	20.0 (7.5-37.5)	22.4 (13.6-43.8)	
Years since quit smoking relative to NMIBC diagnosis (in former smokers)					
≥20	NA	NA	583 (66.7)	NA	NA
10-19	NA	NA	121 (13.8)	NA	
1-9	NA	NA	116 (13.3)	NA	
<1	NA	NA	50 (5.7)	NA	

Abbreviations: BMI, body mass index (calculated as weight in kilograms divided by height in meters squared); CIS, carcinoma in situ; HG, high grade; LG, low grade; NA, not applicable; NMIBC, non–muscle-invasive bladder cancer.

^a^ From Pearson χ^2^ test to compare smoking exposure categories with categorical variables or Fisher exact test for continuous variables.

^b^ Includes American Indian, Alaska Native, or Pacific Islander individuals.

^c^ Rare was defined as drinking a few times per year or once per month; occasional, drinking 2 to 3 times per month, once per week, or twice per week; and regular, drinking 3 to 4 times a week, 5 to 6 times a week, or every day.

^d^ Includes asbestos; chemicals, acids, or solvents; coal or stone dusts, coal tar, pitch, or asphalt; diesel engine exhaust; dyes; formaldehyde; gasoline exhaust; pesticides or herbicides; textile fibers or dusts; wood dust; x-rays or radioactive materials; and smoke other than from cigarettes.

^e^ Includes coal miner; furniture maker; hairdresser; nail salon worker; machinist; painter; printer; truck, taxi, or bus driver; leather industry worker; rubber industry worker; paint industry worker; and textile industry worker.

The mean (SD) time from NMIBC diagnosis to baseline interview was 2.3 (1.3) months. At baseline, 487 participants (33.1%) self-reported as never cigarette smokers, 874 participants (59.4%) reported they were former cigarette smokers, and 111 participants (7.5%) were current cigarette smokers (Table 1). The mean (SD) age at cancer diagnosis was youngest for current smokers (66.9 [9.4] years), followed by never smokers (68.4 [11.8] years) and former smokers (71.6 [10.1] years; *P* < .001). Female participants were more likely to be current smokers (32 participants [28.8%]) than former smokers (183 participants [20.9%]) or never smokers (128 participants [26.3%]; *P* = .03), whereas male participants were more likely to be former smokers (691 participants [79.1%]) than current smokers (79 participants [71.2%]) or never smokers (359 participants [73.7%]; *P* = .03). Former smokers had significantly higher mean (SD) BMI at diagnosis (29.0 [5.6]) compared with never smokers (28.1 [5.7]) and current smokers (27.5 [5.6]; *P* = .001). Current and former smokers were more likely to have been exposed to environmental or chemical exposures compared with never smokers (88 current smokers [79.3%]; 598 former smokers [68.4%]; 314 never smokers [64.5%]; *P* = .009) and more likely to have occupations involving these exposures (47 current smokers [42.3%]; 335 former smokers [38.3%]; 132 never smokers [27.1%]; *P* < .001). Current smokers had smoked for a mean (SD) of 29.5 (23.6) pack-years, while former smokers had smoked for a mean (SD) of 25.6 (23.3) pack-years (Table 1). Two-thirds of former smokers quit smoking at least 20 years before diagnosis (583 participants [66.7%]), and 50 former smokers (5.7%) had quit smoking within 1 year of diagnosis.

Table 2 presents frequencies of self-reported smoking behaviors in the never, former, and current cigarette smokers at baseline. In never cigarette smokers, more than 10% had ever used some other form of smoked tobacco (42 participants [8.6%] had used pipe tobacco and 25 participants [5.1%] had smoked cigars), 86 participants (17.7%) reported ever use of marijuana, and none reported ever use of e-cigarettes. The proportions of pipe smokers in former cigarette smokers (156 participants [17.8%]) and current cigarette smokers (19 participants [17.1%]) were similar, while the proportion of cigar smokers was higher in the former smokers (107 participants [12.2%]) than the current cigarette smokers (12 participants [10.8%]). Currents smokers were more likely to have ever used e-cigarettes (22 participants [19.8%]) or marijuana (45 participants [40.5%]) than former smokers (e-cigarettes: 43 participants [4.9%]; marijuana: 232 participants [26.5%]). Characteristics of participants who reported ever marijuana use are in eTable 2 in Supplement 1.

**Table 2.** Smoking Behaviors by Cigarette Smoking Status in the Be-Well Study

Smoking behavior	No. (%)				*P* value^a^
	Total (n = 1472)	Cigarette smoking status			
		Never (n = 487)	Former (n = 874)	Current (n = 111)	
**Pipe**					
Never	1255 (85.3)	445 (91.4)	718 (82.2)	92 (82.9)	<.001
Ever	217 (14.7)	42 (8.6)	156 (17.8)	19 (17.1)	
**Cigar**					
Never	1328 (90.2)	462 (94.9)	767 (87.8)	99 (89.2)	.001
Ever	54 (9.8)	25 (5.1)	107 (12.2)	12 (10.8)	
**e-Cigarette**					
Never	1407 (95.6)	487 (100)	831 (95.1)	89 (80.2)	<.001
Ever	65 (4.4)	0	43 (4.9)	22 (19.8)	
**Marijuana**					
Never	1107 (75.2)	401 (82.3)	640 (73.2)	66 (59.5)	<.001
Ever	363 (24.7)	86 (17.7)	232 (26.5)	45 (40.5)	
Unknown	2 (0.1)	0	2 (0.2)	0	

^a^ From Pearson χ^2^ test.

Table 3 presents multivariable models of the association of cigarette smoking, other tobacco smoking, e-cigarette use, and marijuana use with risk of recurrence. Over a mean (SD) follow-up period of 25.9 (8.3) months, 473 recurrences, of which 89 were progressions, were confirmed; 136 participants died, and 152 participants were lost to follow-up. Of 89 confirmed progressions, 24 were changes within non–muscle-invasive categories and 65 became muscle-invasive disease. In the minimally adjusted model 1 and compared with less than 10 years, longer duration of cigarette smoking (eg, 10-18 years: HR, 1.57; 95% CI, 1.00-2.46; ≥40 years: HR, 1.96; 95% CI, 1.26-3.06); *P* for trend = .02) and more pack-years (eg, 10-19 pack-years: HR, 1.30; 95% CI, 0.89-1.89; ≥40 pack-years: HR, 1.64; 95% CI, 1.14-2.35; *P* for trend = .02) were associated with higher risk of recurrence. Similarly, in the fully adjusted model 2, increased risk of recurrence was also observed for longer duration (eg, 10-19 years: HR, 1.80; 95% CI, 1.08-3.01; ≥40 years: HR, 2.36; 95% CI, 1.43-3.91; *P* for trend = .004) and pack-years (eg, 10-19 pack-years: 1.41; 95% CI, 0.93-2.15; ≥40 pack-years: HR, 1.97; 95% CI, 1.32-2.95; *P* for trend = .007) (Table 3). None of the other measures of cigarette smoking (ever vs never; never, former, and current; and years since quit) were associated with recurrence. Similarly, no associations with recurrence were observed for pipe, cigar, e-cigarette, or marijuana use (Table 3). There were also no associations of smoking with risk of progression separately (eTable 3 in Supplement 1).

**Table 3.** Associations Between Smoking Behaviors and Risk of Recurrence in the Be-Well Study

Smoking behavior	No.		Recurrence and progression, HR (95% CI) (n = 473)	
	Total	Events	Model 1^a^	Model 2^b^
**Cigarette smoking** ^c^				
Cigarette smoking				
Never	487	159	1 [Reference]	1 [Reference]
Former	874	277	0.93 (0.74-1.15)	0.98 (0.77-1.25)
Current	111	37	1.04 (0.70-1.56)	1.33 (0.85-2.07)
Cigarette smoking duration (in smokers), y				
<10	156	38	1 [Reference]	1 [Reference]
10-19	191	61	1.57 (1.00-2.46)	1.80 (1.08-3.01)
20-29	207	72	1.81 (1.18-2.80)	1.77 (1.07-2.92)
30-39	189	59	1.47 (0.93-2.32)	1.58 (0.94-2.66)
≥40	235	83	1.96 (1.26-3.06)	2.36 (1.43-3.91)
*P* value for trend	NA	NA	.02	.004
Cigarette smoking intensity (in smokers), cigarettes per day				
1-9	204	54	1 [Reference]	1 [Reference]
10-19	236	80	1.28 (0.88-1.87)	1.21 (0.78-1.88)
20-29	344	109	1.19 (0.83-1.71)	1.16 (0.76-1.76)
30-39	91	33	1.40 (0.87-2.24)	1.47 (0.86-2.50)
≥40	102	35	1.24 (0.77-2.00)	1.33 (0.78-2.29)
*P* value for trend	NA	NA	.37	.24
Pack-years (in smokers)				
<10	272	70	1 [Reference]	1 [Reference]
10-19	190	65	1.30 (0.89-1.89)	1.41 (0.93-2.15)
20-29	155	52	1.41 (0.96-2.10)	1.46 (0.93-2.28)
30-39	131	38	1.13 (0.73-1.75)	1.06 (0.65-1.73)
≥40	229	86	1.64 (1.14-2.35)	1.97 (1.32-2.95)
*P* value for trend	NA	NA	.02	.007
Years since quit smoking relative to NMIBC diagnosis (in former smokers)				
≥20	583	173	1 [Reference]	1 [Reference]
10-19	121	44	1.32 (0.92-1.89)	1.35 (0.91-2.03)
1-9	116	42	1.34 (0.91-1.98)	1.47 (0.95-2.28)
<1	50	14	1.02 (0.57-1.84)	1.03 (0.49-2.20)
**Other tobacco and e-cigarette use^d^**				
Pipe				
Never	1255	410	1 [Reference]	1 [Reference]
Ever	217	63	0.86 (0.64-1.17)	0.94 (0.68-1.29)
Cigar				
Never	1328	419	1 [Reference]	1 [Reference]
Ever	144	54	1.26 (0.92-1.72)	1.11 (0.77-1.59)
e-Cigarette				
Never	1407	451	1 [Reference]	1 [Reference]
Ever	65	22	1.12 (0.71-1.75)	0.92 (0.52-1.61)
**Marijuana use^e^**				
Marijuana				
Never	1107	355	1 [Reference]	1 [Reference]
Former	242	76	1.15 (0.87-1.50)	1.03 (0.75-1.40)
Current	121	42	1.03 (0.72-1.48)	0.87 (0.56-1.33)
Marijuana and any tobacco				
No tobacco or marijuana use	358	119	1 [Reference]	1 [Reference]
Tobacco use only	749	236	0.90 (0.70-1.14)	0.96 (0.74-1.26)
Marijuana use only	71	19	0.78 (0.46-1.32)	0.60 (0.31-1.17)
Tobacco and marijuana use	292	99	1.08 (0.81-1.45)	1.02 (0.73-1.43)

Abbreviations: HR, hazard ratio; NA, not applicable; NMIBC, non–muscle-invasive bladder cancer.

^a^ Model 1 was adjusted for age at diagnosis, sex, race and ethnicity, body mass index at diagnosis, cancer stage and grade, chemotherapy, immunotherapy, American Urological Association risk stratification category, educational attainment, and household income.

^b^ Model 2 was adjusted for covariates in model 1 as well as alcohol intake, dietary folate, overall chemical or environmental exposures, and occupation involving chemical or environmental exposures.

^c^ For cigarette smoking, models 1 and 2 also adjusted for ever or never cigar, pipe, marijuana, and e-cigarette use.

^d^ For pipe, cigar, and e-cigarette, models 1 and 2 also adjusted for ever or never cigarette, pipe (if applicable), cigar (if applicable), and e-cigarette use (if applicable).

^e^ For marijuana, models 1 and 2 also adjusted for ever or never cigarette, pipe, cigar, and e-cigarette use, as appropriate.

We examined smoking cessation interventions in 106 Be-Well Study participants at KPNC who were current or recently quit cigarette smokers, among whom 57 received at least 1 smoking cessation intervention and 49 did not receive any intervention (Table 4). Receipt of at least 1 cessation intervention (vs none) did not differ across sociodemographic factors, except for female participants being more likely to receive an intervention than male participants (23 of 30 female participants [76.7%] vs 34 of 76 male participants [44.7%]; *P* = .003). The most common interventions were dispensed prescription medications (51 participants [50.0%]) and wellness coaching (19 participants [19.6%]). Interventions mostly occurred within the 6 months before or after the NMIBC diagnosis (49 participants [48.0%]) or 12 months or later after diagnosis (42 participants [41.2%]).

**Table 4.** Characteristics of Current and Recently Quit Cigarette Smokers Who Were Exposed and Not Exposed to Smoking Cessation Interventions

Characteristic	Participants, No. (%)		*P* value^a^
	Received cessation intervention (n = 57)	Did not receive cessation intervention (n = 49)	
Sex			
Male	34 (59.7)	42 (85.7)	.003
Female	23 (40.4)	7 (14.3)	
Race and ethnicity			
Asian	<5 (1.8)	<5 (2.0)	.17
Black	5 (8.8)	<5 (4.1)	
Hispanic	<5 (3.5)	8 (16.3)	
White	47 (82.5)	36 (73.5)	
Other^b^	<5 (3.5)	<5 (4.1)	
Age group, y			
<50	<5 (3.5)	6 (12.2)	.19
50-64	27 (47.4)	19 (38.8)	
65-79	25 (43.9)	18 (36.7)	
≥80	<5 (5.3)	6 (12.2)	
Mean (SD)	65.7 (9.1)	65.3 (12.4)	.86
Educational attainment			
≤High school or less	17 (29.8)	14 (28.6)	.62
Some college	21 (36.8)	24 (49.0)	
College graduate	13 (22.8)	7 (14.3)	
Postgraduate	5 (8.8)	<5 (8.2)	
Unknown	<5 (1.8)	0	
Marital status			
Never married	6 (10.5)	<5 (2.0)	.05
Married or living as married	27 (47.4)	35 (71.4)	
Separated or divorced	19 (33.3)	9 (18.4)	
Widowed	5 (8.8)	<5 (8.2)	
BMI at diagnosis			
≤24.9	15 (26.3)	14 (28.6)	.93
25.0-29.9	19 (33.3)	17 (34.7)	
≥30.0	23 (40.4)	18 (36.7)	
Household income			
<$60 000	32 (56.1)	14 (28.6)	.03
$60K-$99 999	11 (19.3)	14 (28.6)	
≥$100 000	10 (17.5)	12 (24.5)	
Unknown	<5 (7.0)	9 (18.4)	
Alcohol consumption			
None	13 (22.8)	17 (34.7)	.33
Rare	6 (10.5)	5 (10.2)	
Occasional	9 (15.8)	<5 (6.1)	
Regular	11 (19.3)	11 (22.5)	
Not reported	18 (31.6)	13 (26.5)	

Abbreviations: BMI, body mass index (calculated as weight in kilograms divided by height in meters squared).

^a^ From Pearson χ^2^ test or Fisher exact test.

^b^ Includes American Indian, Alaska Native, or Pacific Islander individuals.

## Discussion

This cohort study included one of the largest samples of patients with NMIBC to date, including 1472 individuals diagnosed and treated in the KPNC and KPSC integrated health care systems. Within this cohort, 59% were former cigarette smokers and 8% were current smokers at cancer diagnosis. Approximately 14% or participants reported ever smoking pipes or cigars only, 4% used e-cigarettes, and 25% used marijuana. Longer duration of cigarette smoking and more pack-years were associated with higher risk of NMIBC recurrence in a dose-dependent manner, with highest risks for participants with 40 years or more of smoking or 40 or more pack-years. No associations of other tobacco, e-cigarette, or marijuana use with recurrence risk were observed. Furthermore, 89 of 473 recurrence events showed progression, but none of the smoking exposures were associated with higher progression risk. We also conducted an exploratory investigation of smoking cessation interventions in the patients treated at KPNC and found varying participation in interventions in the health system around cancer diagnosis, with female patients more likely to engage in an intervention compared with male patients.

Prior studies on prediagnosis smoking and NMIBC recurrence have reported mixed results but had sample size and study design limitations, heterogeneous data collection of smoking behaviors, and no analysis of progression-free survival.^7,8,9,10,11,12,13,14^ A recent study of 723 patients with NMIBC diagnosed between 2014 and 2020 included 83 incident and 640 recurrent cases and found no association of current or former cigarette smoking with recurrence risk.^13^ In addition, several small studies have examined the association of smoking cessation after NMIBC diagnosis and recurrence risk, yet findings have also been mixed with positive^8,30^ and null findings.^31,32^ A 2022 study^33^ of 354 patients with NMIBC diagnosed from 2015 to 2018 reported no association of biochemically verified postdiagnosis smoking with recurrence risk. While we did not specifically investigate postdiagnosis smoking, we found that being a current smoker around the time of diagnosis was not associated with risk of recurrence and progression. To our knowledge, this is the first study to examine the associations of marijuana use with NMIBC prognosis, and we found that marijuana use was not associated with recurrence risk.

Despite no association between being a former or current cigarette smoker at NMIBC diagnosis and worse prognosis, consistent with findings from other studies,^31,32^ we found that increased estimated lifetime duration and pack-years of cigarette smoking were associated with higher risk of recurrence. Given these observations, we hypothesize that years of chronic and cumulative smoking exposure to the bladder urothelium before cancer diagnosis can continue to make the microenvironment susceptible to tumor development. Indeed, several studies have suggested that cancer risk in individuals who quit smoking does not return to that of a never smoker, even though quitting smoking may reduce the risk of developing NMIBC.^5,34,35^ This sustained susceptibility might persist across the disease continuum from before diagnosis to after diagnosis, resulting in elevated risk for recurrence and progression. Thus, cumulative smoking exposure, rather than current smoking status, may be a more meaningful measure to understand the harmful association of smoking with NMIBC prognosis.

Given that 8% of our cohort were current smokers at baseline and the potential aggregate effect of smoking exposure on poor health outcomes, smoking cessation is of high importance in patients with NMIBC. Health care practitioners, especially urologists, can help improve smoking cessation rates during the treatment of patients with NMIBC. Bladder cancer diagnosis and urologist advice have been cited as the most common motivators for cessation in this population.^36,37,38^

At KPNC, a perioperative smoking cessation program began in 2017 as part of standard of care for all patients receiving surgical care, with the goal to reduce risks associated with smoking and surgery.^39,40^ In our exploratory analysis, we found that KPNC Be-Well participants used a variety of available smoking cessation interventions around the time of diagnosis, yet no formal analysis was conducted regarding the association of the intervention with quit rates. Given the initial success of the KPNC perioperative smoking cessation program,^39,40^ we could conduct a comprehensive evaluation of the program focused on patients with NMIBC undergoing surgery using data from the program and the EHR to examine the effectiveness associated with an NMIBC diagnosis and tobacco cessation strategies in recurrence and progression.

This study has several strengths. We used a large sample size with prospective data collection, a contemporary cohort of patients with NMIBC recruited shortly after their cancer diagnosis (mean 2.3 months), collection of detailed self-reported information on tobacco smoking and use of e-cigarettes and marijuana, and complete ascertainment of recurrence and progression outcomes.

### Limitations

This study has some limitations. We cannot rule out recall bias of smoking behavior from participant self-report, and we had a small number of e-cigarette users. Our study findings only apply to the associations of smoking behavior with short-term (≤2 years) and not longer-term risk of recurrence and progression. In the future, we plan to analyze follow-up data on postdiagnosis smoking behavior at 2 years after diagnosis once we have accrued long-term outcome data.

## Conclusions

This prospective cohort study found that longer duration of cigarette smoking and more pack-years were both associated with higher risk of recurrence of NMIBC in a dose-dependent manner. Patients with 40 years or more of smoking had twice the risk of recurrence, with similar risk for those with 40 or more pack-years. Marijuana use was not associated with NMIBC recurrence. These findings suggest that cigarette smoking remains a critical exposure which should be minimized, and early and frequent smoking cessation interventions should be high priority in patients with NMIBC.

## Notes

Supplement 1. **eTable 1.** Be-Well Study Baseline Interview Smoking Questions **eTable 2.** Characteristics of Be-Well Study Participants by Marijuana Smoking Behavior **eTable 3.** Associations Between Smoking Behaviors and Risk of Progression in the Be-Well Study

Supplement 2.
Data Sharing Statement
