# Supplementary material for: Smoking Behaviors and Prognosis in Patients With Non–Muscle-Invasive Bladder Cancer in the Be-Well Study
Source: JAMA Netw Open. 2022 Nov 30;5(11):e2244430. doi: 10.1001/jamanetworkopen.2022.44430 (PMC9713602; doi:10.1001/jamanetworkopen.2022.44430)
Supplement: Supplement 1. — eTable 1. Be-Well Study Baseline Interview Smoking Questions eTable 2. Characteristics of Be-Well Study Participants by Marijuana Smoking Behavior eTable 3. Associations Between Smoking Behaviors and Risk of Progression in the Be-Well Study [file jamanetwopen-e2244430-s001.pdf]

Supplemental Online Content

Kwan ML, Haque R, Young-Wolff KC, et al. Smoking behaviors and prognosis in patients with non–muscle-invasive bladder cancer in the Be-Well study. *JAMA Netw Open*. 2022;5(11):e2244430. doi:10.1001/jamanetworkopen.2022.44430

eTable 1. Be-Well Study Baseline Interview Smoking Questions

eTable 2. Characteristics of Be-Well Study Participants by Marijuana Smoking Behavior

eTable 3. Associations Between Smoking Behaviors and Risk of Progression in the Be-Well Study

This supplemental material has been provided by the authors to give readers additional information about their work.

© 2022 Kwan ML et al. *JAMA Network Open.*

eTable 1. Be-Well Study Baseline Interview Smoking Questions

**1. Have you ever smoked at least 100 cigarettes in your entire life?**	1 ☐ Yes<br>0 ☐ No → **Go to Question 5**<br>8 ☐ Don't Know → **Go to Question 5**<br>9 ☐ Refused → **Go to Question 5**
**2. How old were you when you first started smoking cigarettes on a regular basis (that is at least once/week for three months or more)?**	**|__|__| Age started smoking cigarettes**
**3. Do you currently smoke?**	1 ☐ Yes, every day<br>2 ☐ Yes, some days →<br>**3a. On how many of the past 30 days did you smoke a cigarette?**<br>**|__|__| Days**<br><br>0 ☐ No, not at all →<br>**3b. At what age did you last stop smoking cigarettes?**<br>**|__|__| Age stopped smoking**<br><br>**3c. Excluding all the times you may have quit, how long did you smoke cigarettes? (Years)**<br>**|__|__| Years**<br><br>**3d. Excluding all the times you may have quit, how long did you smoke cigarettes? (Months)**<br>**|__|__| Months**
**4. On average, how many cigarettes do/did you smoke each day?** *(Note to interviewer: 1 pack=20 cigarettes)*	**|__|__|__| Cigarettes per day**

5. Now I am going to ask you some questions about the use of non-cigarette products.

**Product**	**Have you ever used…?**	**If yes…**		
		**Are you currently using…?**	**For how long have you used…?**	**At what age did you begin using…?**
**a. Pipe to smoke tobacco**	1 ☐ Yes<br>0 ☐ No<br>8 ☐ DK<br>9 ☐ Ref	1 ☐ Yes<br>0 ☐ No<br>8 ☐ DK<br>9 ☐ Ref	**|__|__| Years**<br><br>**|__|__| Months**	**|__|__|**

© 2022 Kwan ML et al. *JAMA Network Open.*

Product	Have you ever used…?	If yes…		
		Are you currently using…?	For how long have you used…?	At what age did you begin using…?
**b. Cigars**	1 ☐ Yes<br>0 ☐ No<br>8 ☐ DK<br>9 ☐ Ref	1 ☐ Yes<br>0 ☐ No<br>8 ☐ DK<br>9 ☐ Ref	**|__|__| Years**<br>**|__|__| Months**	**|__|__|**
**c. Chewing tobacco**	1 ☐ Yes<br>0 ☐ No<br>8 ☐ DK<br>9 ☐ Ref	1 ☐ Yes<br>0 ☐ No<br>8 ☐ DK<br>9 ☐ Ref	**|__|__| Years**<br>**|__|__| Months**	**|__|__|**
**d. Marijuana**	1 ☐ Yes<br>0 ☐ No<br>8 ☐ DK<br>9 ☐ Ref	1 ☐ Yes<br>0 ☐ No<br>8 ☐ DK<br>9 ☐ Ref	**|__|__| Years**<br>**|__|__| Months**	**|__|__|**
**e. Snuff**	1 ☐ Yes<br>0 ☐ No<br>8 ☐ DK<br>9 ☐ Ref	1 ☐ Yes<br>0 ☐ No<br>8 ☐ DK<br>9 ☐ Ref	**|__|__| Years**<br>**|__|__| Months**	**|__|__|**

© 2022 Kwan ML et al. *JAMA Network Open.*

Product	Have you ever used…?	If yes…		
		**Are you currently using…?**	**For how long have you used…?**	**At what age did you begin using…?**
**f. Other (e.g., e-cigarettes, snuse, or hookah)**<br><br>**f1. Specify other non-cigarette product______**	1 ☐ Yes<br>0 ☐ No<br>8 ☐ DK<br>9 ☐ Ref	1 ☐ Yes<br>0 ☐ No<br>8 ☐ DK<br>9 ☐ Ref	**|__|__| Years**<br><br>**|__|__| Months**	**|__|__|**

© 2022 Kwan ML et al. *JAMA Network Open.*

eTable 2. Characteristics of Be-Well Study Participants by Marijuana Smoking Behavior

	Never Marijuana Use (n=1,107) n (%)	Ever Marijuana Use (n=363) n (%)	p-value[a]
**Stage/Grade**			
LG Ta	465 (42.0)	166 (45.7)	0.36
LG T1	26 (2.4)	17 (4.7)	
HG Ta	267 (24.1)	85 (23.4)	
HG T1	293 (26.5)	76 (20.9)	
Tis	48 (4.3)	15 (4.1)	
Other	8 (0.7)	4 (1.1)	
**Concomitant CIS**			0.99
No	1,000 (90.3)	329 (90.6)	
Yes with Ta disease	34 (3.1)	12 (3.3)	
Yes with T1 disease	51 (4.6)	17 (4.7)	
CIS only	22 (2.0)	5 (1.4)	
**Chemotherapy (e.g., mitomycin)**			
No	696 (62.9)	198 (54.6)	**0.01**
Yes	411 (37.1)	165 (45.5)	
**Immunotherapy (e.g., BCG)**			
No	522 (47.2)	181 (49.9)	0.69
Yes	585 (52.9)	182 (50.1)	
**Sex**			
Male	815 (73.6)	312 (86.0)	**<0.001**
Female	292 (26.4)	51 (14.0)	
**Race/Ethnicity**			
White	872 (78.8)	292 (80.4)	**0.012**
Black or African American	53 (4.8)	28 (7.7)	
Hispanic	95 (8.6)	32 (8.8)	
Asian	66 (6)	6 (1.7)	
Other (AIAN/PI)	21 (1.9)	5 (1.4)	
**Age Group (y)**			
<50	33 (3)	31 (8.5)	**<0.001**
50-64	209 (18.9)	146 (40.2)	
65-79	595 (53.8)	177 (48.8)	
≥80	270 (24.4)	9 (2.5)	
Mean (SD)	72.2 (10.4)	64.0 (9.7)	**<0.001**
**Body Mass Index at Diagnosis**			
Normal weight (≤24.9)	303 (27.4)	85 (23.4)	0.16
Overweight (25.0-29.9)	448 (40.5)	150 (41.3)	
Obese (≥30.0)	356 (32.2)	128 (35.3)	

© 2022 Kwan ML et al. *JAMA Network Open.*

Mean (SD)	28.5 (5.7)	28.9 (5.7)	0.17
**Marital Status**			
Never married	45 (4.1)	28 (7.7)	**<0.001**
Married or living as married	777 (70.2)	247 (68.0)	
Separated/Divorced	133 (12)	68 (18.7)	
Widowed	143 (12.9)	19 (5.2)	
Unknown	9 (0.8)	1 (0.3)	
**Educational Attainment**			
High school or less	185 (16.7)	65 (17.9)	0.30
Some college	365 (33)	139 (38.3)	
College graduate	303 (27.4)	90 (24.8)	
Post-graduate	241 (21.8)	63 (17.4)	
Unknown	13 (1.2)	6 (1.7)	
**Household Income**			
<$60K	353 (31.9)	104 (28.7)	0.10
$60K-99K	264 (23.9)	82 (22.6)	
≥$100K	349 (31.5)	144 (39.7)	
Unknown	141 (12.7)	33 (9.1)	
**Alcohol Consumption**			
None	269 (24.3)	66 (18.2)	**0.06**
Rare (a few times per year, once per month)	187 (16.9)	49 (13.5)	
Occasional (2-3 times per month, once per week, twice per week)	180 (16.3)	55 (15.2)	
Regular (3-4 times per week, 5-6 times per week, every day)	266 (24)	107 (29.5)	
Unknown (No FFQ available)	205 (18.5)	86 (23.7)	
**Dietary Folate (mcg)**			
Mean (SD)	294.50 (143.21)	325.2 (163.4)	**0.003**
**Overall Exposure to Chemical or Environmental Exposures**[b]			
Never	384 (34.7)	88 (24.2)	**<0.001**
Ever	723 (65.3)	275 (75.8)	
**Occupation Involving Chemical or Environmental Exposures**[c]			
Never	760 (68.7)	197 (54.3)	**<0.001**
Ever	347 (31.4)	166 (45.7)	
**Tobacco Smoking Status**			
Non-tobacco smokers	358 (32.3)	71 (19.6)	**<0.001**
Tobacco Smokers	209 (18.9)	89 (24.5)	
Cigarette Smokers Only	540 (48.8)	203 (55.9)	
**Duration in Years**			
<10 years	n/a	165 (45.5)	n/a
10-19 years	n/a	65 (17.9)	

© 2022 Kwan ML et al. *JAMA Network Open.*

20-29 years	n/a	29 (8.0)	
30-39 years	n/a	37 (10.2)	
>40 years	n/a	62 (17.1)	
Unknown	n/a	5 (1.4)	
Mean (SD, min-max)	n/a	17.0 (16.9, 0-73.0)	
n/a = not applicable			
[a] From Pearson chi-square test to compare smoking exposure categories with categorical variables or Fisher exact test for continuous variables			
[b] Includes asbestos, chemicals/acids/solvents, coal/stone dusts, coal tar/pitch/asphalt, diesel engine exhaust, dyes, formaldehyde, gasoline exhaust, pesticides/herbicides, textile fibers/dusts, wood dust, x-rays/radioactive materials, smoke other than from cigarettes			
[c] Includes coal miner, furniture maker, hairdresser, nail salon worker, machinist, painter, printer, truck/taxi/bus driver, leather industry worker, rubber industry worker, paint industry worker, textile industry worker			

© 2022 Kwan ML et al. *JAMA Network Open.*

eTable 3. Associations Between Smoking Behaviors and Risk of Progression in the Be-Well Study

CIGARETTE SMOKING	Total N	Events N	Progression (n=89)				
			Model 1[a]			Model 2[b c]	
			HR	95% CI		HR	95% CI
**Cigarette Smoking**							
Never	487	34	Ref			Ref	
Ever	985	55	0.75	0.46-1.23		0.85	0.51-1.43
**Cigarette Smoking**							
Never	487	34	Ref			Ref	
Former	874	50	0.76	0.46-1.24		0.84	0.50-1.42
Current	111	5	0.72	0.26-1.99		0.95	0.34-2.65
**Cigarette Smoking Duration (among smokers)**							
1-19 years	353	14	Ref			Ref	
20-39 years	396	26	1.42	0.71-2.83		1.29	0.61-2.70
≥40 years	235	14	1.33	0.59-2.99		1.36	0.57-3.24
p for trend				0.45			0.47
**Cigarette Smoking Intensity (among smokers)**							
1-19 cigarettes	440	21	Ref			Ref	
20-29 cigarettes	344	20	1.02	0.54-1.94		0.97	0.49-1.91
≥30 cigarettes	193	13	1.10	0.52-2.34		1.021	0.45-2.28
p for trend				0.58			0.72
**Pack-Years (among smokers)**							
<20	462	19	Ref			Ref	
20-39	286	21	1.49	0.76-2.93		1.53	0.75-3.12
≥40	229	14	1.15	0.54-2.44		1.11	0.49-2.48
p for trend				0.65			0.73
**Years Since Quit Smoking Relative to NMIBC Diagnosis (among former smokers)**							
Stopped ≥20 years ago	583	30	Ref			Ref	
Stopped 10-19 years ago	121	8	1.20	0.50-2.84		1.16	0.45-2.98
Stopped 1-9 years ago	116	8	1.85	0.79-4.33		1.82	0.73-4.55
Stopped < 1 year ago	50	2	0.71	0.15-3.30		1.04	0.19-5.56
**OTHER TOBACCO USE AND E-CIGARETTE USE**	**Total N**	**Events N**	**Progression (n=89)**				
			**Model 1[a]**			**Model 2[b c]**	
			**HR**	**95% CI**		**HR**	**95% CI**
**Tobacco Smoking (cigarettes, pipes, and/or cigars)**							
Never	429	138	Ref			Ref	
Ever	1,043	335	0.75	0.46-1.23		0.85	0.51-1.43

© 2022 Kwan ML et al. *JAMA Network Open.*

**Pipe Smoking**							
Never	1,255	80	Ref			Ref	
Ever	217	9	0.54	0.26-1.15		0.50	0.22-1.11
**Cigar Smoking**							
Never	1,328	80	Ref			Ref	
Ever	144	9	0.88	0.40-1.92		0.91	0.39-2.13
**E-cigarette Smoking**							
Never	1,407	86	Ref			Ref	
Ever	65	3	1.10	0.33-3.71		1.05	0.24-4.60
**MARIJUANA USE**	**Total N**	**Events N**	**Progression (n=89)**				
			**Model 1[a]**			**Model 2[b c]**	
			**HR**	**95% CI**		HR	95% CI
**Marijuana‡**							
Never	1,107	72	Ref			Ref	
Former	242	12	1.12	0.58-2.15		1.03	0.51-2.08
Current	121	5	0.84	0.33-2.14		0.71	0.24-2.07
**Marijuana and Any Tobacco**							
No tobacco/marijuana use	358	29	Ref			Ref	
Tobacco use only	749	43	**0.56**	**0.33-0.95**		0.65	0.37-1.15
Marijuana use only	71	1	0.22	0.03-1.65		0.28	0.04-2.11
Tobacco and marijuana use	292	16	0.70	0.36-1.36		0.71	0.34-1.47

[a] Model 1: Adjusted for age at diagnosis, sex, race/ethnicity, BMI at diagnosis, stage/grade, chemotherapy, immunotherapy, AUA risk stratification category, educational attainment, and household income

[b] Model 2: Adjusted for covariates in Model 1 + alcohol intake, dietary folate, overall chemical or environmental exposures, occupation involving chemical or environmental exposures

[c] For cigarette smoking, Models 1 and 2 also adjusted for ever/never cigar and pipe, marijuana, and e-cigarettes. For pipe, cigar, and e-cigarette, Models 1 and 2 also adjusted for ever/never cigarette, pipe (if applicable), cigar (if applicable), and e-cigarettes (if applicable). For marijuana, Models 1 and 2 also adjusted for ever/never cigarette, pipe, cigar, and e-cigarettes as appropriate.

© 2022 Kwan ML et al. *JAMA Network Open.*
