# Supplementary material for: Smoking Behaviors and Prognosis in Patients With Non–Muscle-Invasive Bladder Cancer in the Be-Well Study
Source: JAMA Netw Open. 2022 Nov 30;5(11):e2244430. doi: 10.1001/jamanetworkopen.2022.44430 (PMC9713602; doi:10.1001/jamanetworkopen.2022.44430)
Supplement: Supplement 2. — Data Sharing Statement [file jamanetwopen-e2244430-s002.pdf]

Data Sharing Statement

Kwan. Smoking Behaviors and Prognosis in Patients With Non-Muscle-Invasive Bladder Cancer in the Be-Well Study. *JAMA Netw Open*. Published November 30, 2022. doi:10.1001/jamanetworkopen.2022.44430

Data
Data available: No

Additional Information
Explanation for why data not available: Any interested individuals are welcome to contact us for further discussion.
